# Central nervous system prophylaxis with intrathecal liposomal cytarabine in a subset of high-risk patients with diffuse large B-cell lymphoma receiving first line systemic therapy in a prospective trial

**DOI:** 10.1007/s00277-016-2648-4

**Published:** 2016-03-30

**Authors:** E. González-Barca, M. Canales, A. Salar, J. J. Ferreiro-Martínez, S. Ferrer-Bordes, J. A. García-Marco, J. J. Sánchez-Blanco, J. García-Frade, J. Peñalver, J. L. Bello-López, J. M. Sancho, D. Caballero, on behalf of the GELTAMO group

**Affiliations:** Institut Català d’Oncologia. IDIBELL., Hospital Duran i Reynals, Av. Gran Vía 199-203, 08908 L’Hospitalet de Llobregat, Barcelona, Spain; Hospital Universitario La Paz, Madrid, Spain; Hospital del Mar, Barcelona, Spain; Hospital Donostia, San Sebastián, Spain; Hospital Dr. Peset, Valencia, Spain; Hospital Puerta de Hierro, Madrid, Spain; Hospital Morales Meseguer, Murcia, Spain; Hospital Río Hortega, Valladolid, Spain; Fundación Hospital de Alcorcón, Madrid, Spain; Complexo Hospitalario de Santiago, Santiago de Compostela, Spain; Institut Català d’Oncologia - Hospital Germans Trias i Pujol, Badalona, Spain; Hospital Clínico Universitario de Salamanca, Salamanca, Spain

**Keywords:** Central nervous system, Prophylaxis, Liposomal cytarabine, Diffuse large B-cell lymphoma

## Abstract

The dissemination in the central nervous system (CNS) is an uncommon but fatal complication occurring in patients with diffuse large B-cell lymphoma (DLBCL). Standard prophylaxis has been demonstrated to reduce CNS relapse and improve survival rates. Intrathecal (IT) liposomal cytarabine allows maintaining elevated drug levels in the cerebrospinal fluid for an extended period of time. Data on the efficacy and safety of liposomal cytarabine as CNS prophylaxis in patients with DLBCL are still insufficient. The objective of the present study was to evaluate the effectiveness and safety of the prophylaxis with IT liposomal cytarabine in prevention of CNS relapse in high-risk patients with DLBCL who were included in a trial of first line systemic therapy with 6 cycles of dose-dense R-CHOP every 14 days. Twenty-four (18.6 %) out of 129 patients were identified to have risk factors for CNS involvement, defined as follows: >30 % bone marrow infiltration, testes infiltration, retroperitoneal mass ≥10 cm, Waldeyer ring, or bulky cervical nodes involvement. Liposomal cytarabine (50 mg) was administered by lumbar puncture the first day of the 1st, 2nd, and 6th cycle of R-CHOP14 scheme. Among 70 IT infusions, grade 3–4 adverse events reported were headache (one patient) and nausea/vomiting (one patient). With a median follow-up of 40.1 months, no CNS involvement by DLBCL was observed in any patient. In conclusion, IT liposomal cytarabine is safe, feasible, and effective for CNS prophylaxis, causing few associated risks and little discomfort to patients with DLBCL.

## Introduction

Diffuse large B-cell lymphoma (DLBCL) is the most common type of non-Hodgkin’s lymphoma in adults, presenting an annual incidence estimated in 3–5 cases per 100,000 inhabitants [1, 2]. The gold standard treatment for DLBCL includes rituximab, cyclophosphamide, doxorubicin, vincristine, and prednisone (R-CHOP) administered every 14 or 21 days, achieving long-term disease-free survival in approximately 60 % of patients [3–6]. The dissemination in the central nervous system (CNS) is an uncommon complication with an incidence rate of about 5 %, but can rise up to 25 % depending on patient’s risk factors, such as elevated serum lactate dehydrogenase (LDH) or the implication of extranodal sites or testes [7–14]. CNS involvement occurs early in the course of the DLBCL (4.7–9.0 months from diagnosis) and is associated with poor outcomes since survival drops to 2–5 months. Prophylaxis has been demonstrated to reduce CNS relapse and improve survival rates [15, 16]. For this reason, American and European guidelines recommend the performance of a diagnostic lumbar puncture and the administration of CNS prophylaxis for high-risk patients [17, 18]. Standard strategy for the prophylaxis includes systemic chemotherapy that crosses hematoencephalic barrier, intrathecal (IT) chemotherapy, radiation therapy, or their combination [19]. IT chemotherapy has become the approach most frequently used because acting directly in the cerebrospinal fluid (CSF) and maintaining therapeutic drug concentrations [20, 21]. Main IT regimens include methotrexate (MTX) or cytarabine. Liposomal cytarabine, a sustained-release preparation of cytarabine for IT administration, allows maintaining elevated drug levels in the CSF for an extended period of time (>14 days) [19, 22]. The efficacy and safety of liposomal cytarabine have been demonstrated in Burkitt’s lymphoma, acute lymphoblastic leukemia, and unclassifiable highly aggressive B-cell lymphoma [23, 24]. However, data in patients with DLBCL in prophylactic therapy are still insufficient.

The objective of the present study was to evaluate the effectiveness and safety of the CNS prophylaxis with IT liposomal cytarabine in high-risk patients with DLBCL who were included in a prospective trial of first line systemic therapy with 6 cycles of dose-dense R-CHOP every 14 days.

## Patient and methods

### Study population

A single arm open label multicentre study conducted between 2006 and 2011 evaluated dose-dense R-CHOP every 14 days in DLBCL patients (EudraCT identifier 2005-005110-20). The criteria for inclusion were as follows: aged over 18, diagnosis of DLBCL [25], Eastern Cooperative Oncology Group performance status (ECOG PS) 0–2, any International Prognostic Index (IPI) if aged over 65, or IPI 0–2 (if under 65), B-cells positive for CD20, and the informed consent signed. Among the exclusion criteria were pregnancy or breastfeeding, central nervous system lymphoma, severe impairment of the renal function, human immunodeficiency virus (HIV) infection, previous treatment for DLBCL, heart failure (ejection fraction <40 %), severe psychiatric disorders, and hypersensitivity to murine proteins or any other component of the drug. The present study included a subset of patients who required CNS prophylaxis with liposomal cytarabine (DepoCyt®) by presenting one or more of the following risk factors for CNS involvement (considered when the trial was designed): > 30 % bone marrow infiltration, testes infiltration, retroperitoneal mass ≥10 cm, Waldeyer ring involvement, or bulky cervical nodes involvement.

### Experimental procedure

**Fig. 1:**
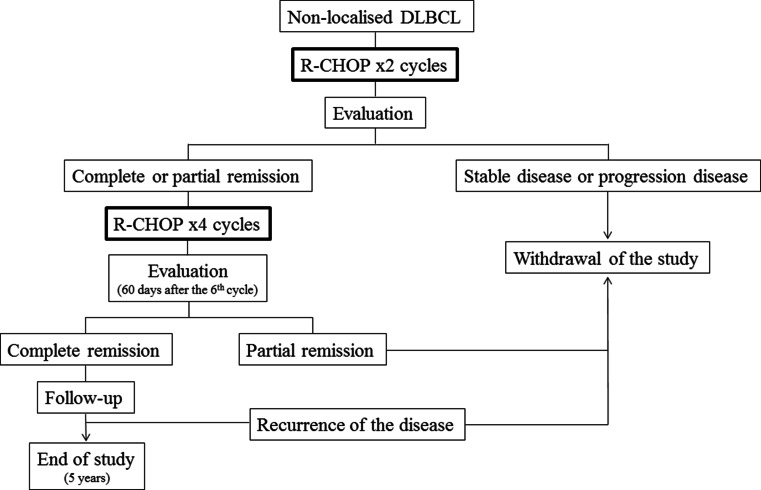
Scheme of R-CHOP cycles and evaluation times during the study

R-CHOP was administered every 14 days for 6 cycles (R-CHOP14): 375 mg/m^2^ rituximab iv on day 1 plus CHOP chemotherapy regimen (750 mg/m^2^ cyclophosphamide iv + 50 mg/m^2^ adriamycin iv + 1.4 mg/m^2^ vincristine iv (maximal dose 2 mg), all on day 1 plus 100 mg oral prednisone on days 1–5). At day 2 of each cycle, 6 mg pegfilgrastim was administered subcutaneously. The tumor response, i.e., complete remission (CR), partial remission (PR), stable disease (SD), and progression disease (PD), was evaluated according to standardized response criteria for non-Hodgkin’s lymphoma [26]. Clinical response was evaluated after the 2nd cycle of treatment. In case of achieving CR or PR, the patient continued with four additional cycles, and the response was evaluated within 60 days after finishing the 6th cycle. Clinical follow-up was performed every 3 months, in the first and second year, and every 6 months in the following years. Criteria for withdrawal of the study included no response or PD after the 2nd cycle, partial remission after the 6th cycle, recurrence of the lymphoma, unacceptable toxicity, or refusal to continue with the treatment (Fig. 1). Liposomal cytarabine (50 mg) was administered by lumbar puncture on day 1 of the 1st, 2nd, and 6th cycle of R-CHOP14. Patients also received the concurrent administration of 4 mg dexamethasone (intrathecal or oral). CNS involvement was assessed by using standard CSF cytology or flow cytometry. Procedures were performed in accordance with the guidelines established in the Declaration of Helsinki. Toxicity due to IT liposomal cytarabine was determined according to Common Terminology Criteria for Adverse Events (AEs), version 3.0. Secondary endpoints included the evaluation of the overall survival (OS), time to progression or relapse, and progression-free survival (PFS). The analysis was performed using the intended-to-treat population. Survival functions were estimated by using Kaplan–Meier method (95 % confidence interval, 95 % CI). All the statistical procedures were performed using SAS 9.0.

## Results

### Characteristics of high-risk patients for CNS involvement

From 129 patients of the multicenter study, 24 (18.6 %) cases presented at least one of the risk factors for CNS involvement considered when the trial was designed. Demographic, clinical characteristics, and risk factors for CNS involvement of this subset of patients at diagnosis are shown in Table 1. Median time from diagnosis to IT prophylaxis was 0.5 months (IQR, 48.0–72.5).

**Table 1:** Demographic and clinical characteristics of the 24 patients with risk factors for CNS involvement at diagnosis

	Number	Percentage
Median age (limits)	66.5	18–80
Sex male	16	66.7
ECOG		
0	13	54.2
1	9	37.5
2	2	8.3
IPI		
1–2	17	70.8
3–4	7	29.2
B symptoms	5	20.8
Ann Arbor stage		
I–II	11	45.8
III–IV	13	54.2
Bulky disease	7	29.2
Elevated LDH	11	45.8
Extranodal involvement	16	66.7
≥2 sites	4	16.7
Risk factors for CNS prophylaxis		
Testes involvement	2	8.3
Infiltration of bone marrow (>30 %)	2	8.3
Waldeyer ring involvement	6	25.0
Retroperitoneal mass ≥10 cm	7	29.1
Bulky cervical nodes involvement	8	33.3
Hollender criteria for risk of CNS involvement (albumin levels not available)		
0	4	16.7
1	10	41.7
2	5	20.8
3	4	17.7
4	1	4.2
Schmitz/Savage criteria for risk of CNS involvement		
1 factor	8	33.3
2–3 factors	12	50.0
4–6 factors	4	16.7
Other risk factors for CNS involvement		
Elevated LDH and ≥2 extranodal involvement	2	8.3
Elevated LDH and Ann Arbor stage III–IV	7	29.0
Kidney/adrenal gland involvement	2	8.3
Breast involvement	0	0.0

*CNS* central nervous system, *ECOG* Eastern Cooperative Oncology Group performance status, *IPI* International Prognostic Index, *LDH* lactate dehydrogenase

### Clinical outcomes among the 24 patients with high risk for CNS involvement

**Fig. 2:**
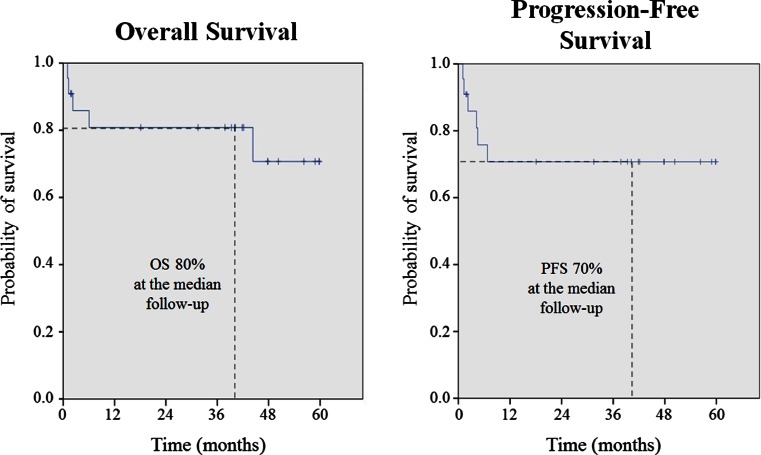
Analysis of the overall survival, time to progression/relapse, and progression-free survival achieved by R-CHOP treatment

From the 24 patients, 21 were evaluable for response 60 day after the 6th R-CHOP, 18 patients (75 %) achieved CR and 1 (4 %) PR. With a median follow-up of 40.1 months, 3 year OS was 80.8 % (95 % CI, 63.8–97.8), and 3 year PFS was 70.7 % (95 % CI 50.9–90.5) (Fig. 2). One patient (4.2 %) relapsed, and three (12.5 %) patients progressed. None of the patients experienced CNS relapse during the follow-up period. The patient who relapsed developed a mediastinal mass. Six patients (25.0 %) died during the follow-up due to lung cancer (*n* = 1), pneumonia (*n* = 1), disease progression (*n* = 2), sepsis by *Escherichia coli* (*n* = 1), and unknown cause (*n* = 1).

### Prophylactic effect of intrathecal liposomal cytarabine

The analysis of the CSF was performed by cytology in 52 samples and by flow cytometry in 7 samples. CSF was negative for lymphoma infiltration at diagnosis in all patients. Adverse events of liposomal cytarabine intrathecal therapy among 70 IT infusions are shown in Table 2. A total of 18 patients (75.0 %) completed the three doses of IT liposomal cytarabine. Causes of discontinuation were as follows: toxicity (*n* = 1), systemic progression (*n* = 1), medical decision (*n* = 1), change to methylprednisolone treatment (*n* = 1), or death (2). Most of IT infusions 64/70 (91.4 %) were with concurrent administration of dexamethasone.

**Table 2:** Adverse events of 70 liposomal cytarabine IT infusions for CNS prophylaxis

	Grade 1–2	Grade 3–4
Headache	2	1
Dizziness	1	
Confusion	1	
Nausea/vomiting		1

## Discussion

The implication of the CNS is an uncommon complication with very poor prognosis occurring in patients with DLBCL [7]. While the addition of rituximab to CHOP regimen has demonstrated to improve clinical outcomes in patients with DLBCL, its effect on CNS dissemination is unclear [27]. CNS prophylaxis has become a standard procedure recommended in high-risk patients since the demonstration of reducing CNS relapse and improving survival rates [15, 16]. The identification of risk factors at diagnosis for CNS relapse in DLBCL patients is a controversial issue. Hollender et al. described five risk factors in the pre-rituximab era: older than 60 years, elevated LDH, low albumin levels, two or more extranodal involvement, and bulky retroperitoneal mass [28]. In recent years, Schmitz et al., basing on data from the MiNT trial, have found that the optimal risk model included the combination of the involvement of more than one extranodal site and elevated levels of LDH [29]. When the analysis was restricted to patients receiving rituximab with chemotherapy, the risk model included advanced stage and elevated LDH. Savage et al. have recently confirmed the prognostic model proposed by the German group, which includes the five risk factors of IPI in addition to kidney/adrenal gland involvement [30, 31], in a large cohort of DLBCL patients. Furthermore, certain extranodal sites such as testis [32], breast [33], and kidney [34] have also been considered to increase the risk of CNS progression [35–37]. In our study, we analyzed retrospectively the risk factors identified in the rituximab era as shown in Table 1. Almost one third of the patients had simultaneously advanced stage and elevated LDH. At the time that our trial was designed, patients with known factors for a higher risk of CNS progression were included for CNS prophylaxis.

There are different strategies to prevent CNS lymphoma involvement in high-risk patients. One of them consists on high dose iv methotrexate (3.0–3.5 g/m^2^) alternating with chemotherapy [38–40]. This is an effective option; however, it can be only used in young patients due to its higher toxicity, and it also may cause the delay of the systemic therapy. The use of IT injections of antineoplastic drugs is another option for CNS involvement prophylaxis. Among the different IT prophylactic strategies, liposomal cytarabine allows maintaining cytotoxic levels of the free drug in the CSF for an extended period of time (>14 days) [19, 22]. The administration of IT liposomal cytarabine is less frequent than conventional therapies, leading to minimize the patient’s discomfort and risks associated with lumbar puncture procedures. Randomized clinical trials have demonstrated similar efficacy of IT liposomal cytarabine as IT MTX or conventional cytarabine in patients with leptomeningeal infiltration [23, 41, 42]. Furthermore, in comparison with IT cytarabine, liposomal cytarabine has demonstrated higher response rates and a significant improvement in quality of life [43]. The efficacy of liposomal cytarabine has been demonstrated in Burkitt’s lymphoma, acute lymphoblastic leukemia, and unclassifiable highly aggressive B-cell lymphoma [23, 24]. However, there are no data on patients with DLBCL for prophylactic purposes. Although only 24 patients were treated in our study with prophylactic IT liposomal cytarabine, none of them developed CNS relapse during a follow-up period of 40.1 months.

Our scheme of liposomal cytarabine administration (at 1st, 2nd, and 6th cycle of R-CHOP14) was designed to reduce half of the injections of the standard IT therapies, and taking into account that the risk of CNS involvement is higher during the first cycles, when the tumor is more active, leaving the last dose as consolidation. It has been demonstrated in this trial that the scheme is well tolerated and safe. Clinical studies evaluating safety of IT liposomal cytarabine in patients with DLBCL are insufficient. Most of the safety data of IT liposomal cytarabine have been reported in highly pre-treated patients with CNS involvement and with other aggressive lymphoproliferative diseases, such as Burkitt lymphoma and lymphoblastic leukemia. Commonly described AEs associated with liposomal cytarabine include headache, nausea, fever, vomiting, and back pain. Reported grade 3–4 AEs include arachnoiditis, persistent lumbar pain, peripheral sensory neuropathy, motor neuropathy, and optical nerve neuritis [44–47]. On the other hand, one study has reported severe neurotoxicity (including seizures, encephalitis, cauda equina syndrome, or pseudotumor cerebri) associated with IT liposomal cytarabine administered concurrently with high-dose MTX and cytarabine in patients with acute lymphocytic leukemia [48]. Therefore, the concurrent administration of these drugs is contraindicated. Patients in our study were receiving their first line systemic therapy and prophylactic IT liposomal cytarabine, and toxicity has been much lower, 25 % of patients suffered IT chemotherapy related events, and only 8.3 % suffered grade 3/4 AEs. Patients experienced grade 1–2 headache (*n* = 2), dizziness (*n* = 1), confusion (*n* = 1), and grade 3–4 headache (*n* = 1) and nausea/vomiting (*n* = 1). In a previous study of our group, Garcia-Marco et al. evaluated retrospectively the efficacy and safety of the therapeutic use of liposomal cytarabine for the treatment of lymphomatous meningitis in patients with lymphoma, mainly DLBCL [49]. Fifty patients received corticosteroids for the prevention of chemical arachnoiditis. The most commonly used agent was dexamethasone (usually 4 mg po twice daily for 5 days with each cycle of liposomal cytarabine). Liposomal cytarabine was well tolerated and produced no AEs in 30 out of 54 of the patients. In the remaining ones, headache grade 1–2 (*n* = 17) was the most common AE reported, followed of nausea (*n* = 7), fever (*n* = 7), vomiting (*n* = 6), neurologic deficits (*n* = 2), and dizziness (*n* = 1). By contrast with our study, one patient (receiving also dexamethasone) did have neurotoxicity. Recently, Krawczyk et al. also determined efficacy and safety of liposomal cytarabine as CNS prophylaxis in patients with DLBCL [50]. All patients received oral prednisone (as an element of the R-CHOP regimen) on administration of intrathecal liposomal cytarabine. In their study, 59 out of 79 patients (74.7 %) experienced AEs, mainly headache. Other AEs were nausea (*n* = 13), fever (*n* = 10), vomiting (*n* = 5), dizziness (*n* = 3), neurological deficits (*n* = 3), and myelopathy (*n* = 1). The rate of AEs was higher than in our study, as seven patients suffered grade 3–4. In another study of our group, 54 patients with lymphoma (25 DLBCL) received prophylactic IT cytarabine with 4 mg IT dexamethasone and 20 mg iv dexamethasone on day 1, and only 4 (3.5 %) grade 3 AEs among 112 administrations were observed. Therefore, it seems that the concurrent administration of dexamethasone can avoid the development of chemical arachnoiditis [51]. The main limitation of the present study was the low number of high-risk patients available in the original trial. Nevertheless, it is important to highlight that our results are derived from a prospective study defined by a homogeneous systemic treatment in all the patients.

In conclusion, IT liposomal cytarabine has demonstrated being safe, feasible, and effective for CNS prophylaxis, causing few associated risks and little discomfort to patients with DLBCL.

## References

[1] Møller MB, Pedersen NT, Christensen BE (2004). Diffuse large B-cell lymphoma: clinical implications of extranodal versus nodal presentation—a population-based study of 1575 cases. Br J Haematol.

[2] Martelli M, Ferreri AJ, Agostinelli C (2013). Diffuse large B-cell lymphoma. Crit Rev Oncol Hematol.

[3] Coiffier B, Lepage E, Briere J (2002). CHOP chemotherapy plus rituximab compared with CHOP alone in elderly patients with diffuse large-B-cell lymphoma. N Engl J Med.

[4] Feugier P, Van HA, Sebban C (2005). Long-term results of the R-CHOP study in the treatment of elderly patients with diffuse large B-cell lymphoma: a study by the Groupe d’Etude des Lymphomes de l’Adulte. J Clin Oncol.

[5] Pfreundschuh M, Trumper L, Osterborg A (2006). CHOP-like chemotherapy plus rituximab versus CHOP-like chemotherapy alone in young patients with good-prognosis diffuse large-B-cell lymphoma: a randomised controlled trial by the MabThera International Trial (MInT) Group. Lancet Oncol.

[6] Cunningham D, Hawkes EA, Jack A (2013). Rituximab plus cyclophosphamide, doxorubicin, vincristine, and prednisolone in patients with newly diagnosed diffuse large B-cell non-Hodgkin lymphoma: a phase 3 comparison of dose intensification with 14-day versus 21-day cycles. Lancet.

[7] Bierman P, Giglio P (2005). Diagnosis and treatment of central nervous system involvement in non-Hodgkin’s lymphoma. Hematol Oncol Clin North Am.

[8] Hegde U, Filie A, Little RF (2005). High incidence of occult leptomeningeal disease detected by flow cytometry in newly diagnosed aggressive B-cell lymphomas at risk for central nervous system involvement: the role of flow cytometry versus cytology. Blood.

[9] Boehme V, Zeynalova S, Kloess M (2007). Incidence and risk factors of central nervous system recurrence in aggressive lymphoma—a survey of 1693 patients treated in protocols of the German High-Grade Non-Hodgkin’s Lymphoma Study Group (DSHNHL). Ann Oncol.

[10] Bjorkholm M, Hagberg H, Holte H (2007). Central nervous system occurrence in elderly patients with aggressive lymphoma and a long-term follow-up. Ann Oncol.

[11] Boehme V, Schmitz N, Zeynalova S, Loeffler M, Pfreundschuh M (2009). CNS events in elderly patients with aggressive lymphoma treated with modern chemotherapy (CHOP-14) with or without rituximab: an analysis of patients treated in the RICOVER-60 trial of the German High-Grade Non-Hodgkin Lymphoma Study Group (DSHNHL). Blood.

[12] Shimazu Y, Notohara K, Ueda Y (2009). Diffuse large B-cell lymphoma with central nervous system relapse: prognosis and risk factors according to retrospective analysis from a single-center experience. Int J Hematol.

[13] Bernstein SH, Unger JM, Leblanc M, Friedberg J, Miller TP, Fisher RI (2009). Natural history of CNS relapse in patients with aggressive non-Hodgkin’s lymphoma: a 20-year follow-up analysis of SWOG 8516—the Southwest Oncology Group. J Clin Oncol.

[14] Korfel A (2011). Prevention of central nervous system relapses in diffuse large B-cell lymphoma: which patients and how?. Curr Opin Oncol.

[15] Cortes J, O’Brien S, Pierce S, Keating MJ, Freireich EJ, Kantarjian H (1995). The value of high-dose systemic chemotherapy and intrathecal therapy for central nervous system prophylaxis in different risk groups of adult acute lymphoblastic leukemia. Blood.

[16] Blum K, Lozanski G, Byrd J (2004). Adult Burkitt leukemia and lymphoma. Blood.

[17] National Comprehensive Cancer Network (2010) National Comprehensive Cancer Network guidelines for treatment of cancer by site: non-Hodgkin’s lymphoma. http://www.nccn.org/professionals/physician_gls/f_guidelines.asp. Accessed Dec 19 2014

[18] Tilly H, Dreyling M (2010). Diffuse large B-cell non-Hodgkin’s lymphoma: ESMO Clinical Practice Guidelines for diagnosis, treatment and follow-up. Ann Oncol.

[19] Jabbour E, Thomas D, Cortes J, Kantarjian HM, O’Brien S (2010). Central nervous system prophylaxis in adults with acute lymphoblastic leukemia: current and emerging therapies. Cancer.

[20] Cortes J, O’Brien SM, Pierce S, Keating MJ, Freireich EJ, Kantarjian HM (1995). The value of high-dose systemic chemotherapy and intrathecal therapy for central nervous system prophylaxis in different risk groups of adult acute lymphoblastic leukemia. Blood.

[21] DeAngelis L (2004). Primary CNS lymphoma: is there a role for prophylaxis against lymphomatous meningitis?. Expert Rev Neurother.

[22] Kim S, Chatelut E, Kim J (1993). Extended CSF cytarabine exposure following intrathecal administration of DTC 101. J Clin Oncol.

[23] Glantz MJ, LaFollette S, Jaeckle KA (1999). Randomized trial of a slow-release versus a standard formulation of cytarabine for the intrathecal treatment of lymphomatous meningitis. J Clin Oncol.

[24] Cortelazzo S, Ponzoni M, Ferreri AJ, Hoelzer D (2011). Lymphoblastic lymphoma. Crit Rev Oncol Hematol.

[25] Aisenberg AC (1995). Coherent view of non-Hodgkin’s lymphoma. J Clin Oncol.

[26] Cheson BD, Horning SJ, Coiffier B (1999). Report of an international workshop to standardize response criteria for non-Hodgkin’s lymphoma. J Clin Oncol.

[27] Guirguis HR, Cheung MC, Mahrous M (2012). Impact of central nervous system (CNS) prophylaxis on the incidence and risk factors for CNS relapse in patients with diffuse large B-cell lymphoma treated in the rituximab era: a single centre experience and review of the literature. Br J Haematol.

[28] Hollender A, Kvaloy S, Nome O (2002). Central nervous system involvement following diagnosis of non-Hodgkin lymphoma: a risk model. Ann Oncol.

[29] Schmitz N, Zynalova S, Galss B (2012). CNS disease in younger patients with aggressive B-cell lymphoma: an analysis of patients treated on the Mabthera International Trial and trials of the German High-Grade Non-Hodgkin Lymphoma Study Group. Ann Oncol.

[30] Schmitz N, Zeylaniva S, Nikelsen M (2013). A new prognostic model to assess the risk of CNS disease in patients with aggressive B-cell lymphoma. Hematol Oncol.

[31] Savage KJ, Samira Zeynalova S, Kansara RR (2014). Validation of a prognostic model to assess the risk of CNS disease in patients with aggressive B-Cell lymphoma. Blood.

[32] Zucca E, Conconi A, Mughal TI (2003). Patterns of outcome and prognostic factors in primary large-cell lymphoma of the testis in a survey by the International Extranodal Lymphoma Study Group. J Clin Oncol.

[33] Hosein PJ, Maragulia JC, Salzberg MP (2014). A multicentre study of primary breast diffuse large B-cell lymphoma in the rituximab era. Br J Haematol.

[34] Villa D, Connors JM, Sehn LH, Gascoyne RD, Savage KJ (2011). Diffuse large B-cell lymphoma with involvement of the kidney: outcome and risk of central nervous system relapse. Haematologica.

[35] Fletcher CD, Kahl BS (2014). Central nervous system involvement in diffuse large B-cell lymphoma: an analysis of risk and prevention strategies in the post-rituximab era. Leuk Lymphoma.

[36] Ferreri AJ, Bruno-Ventre M, Donadoni G (2015). Risk tailored CNS prophylaxis in a mono-institutional series of 200 patients with diffuse large B-cell lymphoma treated in the rituximab era. Br J Haematol.

[37] Sancho JM, Ribera, JM (2015) Central nervous system relapse in diffuse large B-cell lymphoma: risk factors. Med Clin (Barc) 10.1016/j.medcli.2014.12.025 25817451

[38] Abramson JS, Hellmann M, Barnes JA (2010). Intravenous methotrexate as central nervous system (CNS) prophylaxis is associated with a low risk of CNS recurrence in high-risk patients with diffuse large B-cell lymphoma. Cancer.

[39] Récher C, Coiffier B, Haioun C (2011). Intensified chemotherapy with ACVBP plus rituximab versus standard CHOP plus rituximab for the treatment of diffuse large B-cell lymphoma (LNH03-2B): an open-label randomised phase 3 trial. Lancet.

[40] Holte H, Leppä S, Björkholm M (2013). Dose-densified chemoimmunotherapy followed by systemic central nervous system prophylaxis for younger high-risk diffuse large B-cell/follicular grade 3 lymphoma patients: results of a phase II Nordic lymphoma group study. Ann Oncol.

[41] Howell SB (2003). Liposomal cytarabine for the treatment of lymphomatous meningitis. Biol Ther Lymphoma.

[42] Benesch M, Urban C (2008). Liposomal cytarabine for leukemic and lymphomatous meningitis: recent developments. Expert Opin Pharmacother.

[43] Cole BF, Glantz MJ, Jaeckle KA, Chamberlain MC, Mackowiak JI (2003). Quality-of-life-adjusted survival comparison of sustained-release cytosine arabinoside versus intrathecal methotrexate for treatment of solid tumor neoplastic meningitis. Cancer.

[44] Pacira Pharmaceuticals (2007). DepoCyt (liposomal cytarabine) prescribing information.

[45] Gökbuget N, Hartog CM, Bassan R (2011). Liposomal cytarabine is effective and tolerable in the treatment of central nervous system relapse of acute lymphoblastic leukemia and very aggressive lymphoma. Haematologica.

[46] Corazzelli G, Frigeri F, Russo F (2012). RD-CODOX-M/IVAC with rituximab and intrathecal liposomal cytarabine in adultBurkitt lymphoma and ‘unclassifiable’ highly aggressive B-cell lymphoma. Br J Haematol.

[47] Segot A, Raffoux E, Lengline E (2015). Liposomal cytarabine in prophylaxis or curative treatment of central nervous system involvement in Burkitt leukemia/lymphoma. Ann Hematol.

[48] Jabbour E, O’Brien S, Kantarjian H (2007). Neurologic complications associated with intrathecal liposomal cytarabine given prophylactically in combination with high-dose methotrexate and cytarabine to patients with acute lymphocytic leukemia. Blood.

[49] Garcia-Marco JA, Panizo C, Garcia ES (2009). Efficacy and safety of liposomal cytarabine in lymphoma patients with central nervous system involvement from lymphoma. Cancer.

[50] Krawczyk K, Jurczak W, Długosz-Danecka M (2013). Central nervous system prophylaxis with intrathecal liposomal cytarabine in diffuse large B-cell lymphomas. Pol Arch Med Wewn.

[51] De la Fuente A, Olave M, Peñalver FJ (2014). DEPO-ITV: a new scenario with intrathecal and intravenous dexamethasone to prevent chemical arachnoiditis related with liposomal cytarabine. Haematologica.

